# The association between Internet use and health-related outcomes in older adults and the elderly: a cross-sectional study

**DOI:** 10.1186/s12911-021-01500-2

**Published:** 2021-05-06

**Authors:** Mariusz Duplaga

**Affiliations:** Department of Health Promotion and e-Health, Institute of Public Health, Faculty of Health Sciences, Jagiellonian University Medical College, Skawińska Str. 8, 31-066 Kraków, Poland

**Keywords:** Internet use, Elderly, Older people, Health behaviours, Health literacy, Self-assessment of health status, Utilisation of health services

## Abstract

**Background:**

Older adults and elderly persons can experience a significant digital divide. However, there are many studies reporting that the use of the Internet has benefits related to a person’s health status and social inclusion. It is not fully clear if Internet use and health-related outcomes share common antecedents or if using the Internet may have a favourable effect on the determinants responsible for good health. The main aim of this study was to assess the relationship between the use of the Internet and health-related outcomes in older adults and elderly people in Poland.

**Methods:**

The effect of the use of the Internet was analysed with regression models after adjusting for sociodemographic factors. The data used for the analysis were obtained through a telephone-based survey on a representative sample of Polish adults at least 50 years of age.

**Results:**

By categorising the frequency of Internet use by the respondents, it was found that some categories were significantly related to less favourable health behaviours. Rare Internet users were less likely to undertake physical activity than nonusers (odds ratio, OR, 95% confidence interval, 95%CI 0.48, 0.28–0.83). Those using the Internet every day less frequently consumed fruit and vegetables (OR, 95%CI 0.64, 0.42–0.99). Internet use was also associated with more frequent consumption of alcohol (OR, 95%CI 1.65, 1.09–2.50 comparing nonusers with those accessing the Internet several times a week). Persons rarely using the Internet, but not those who accessed it at least a few times a week, possessed a lower health literacy than nonusers (OR, 95%CI 0.71, 0.52–0.97). Internet users were also more likely to assess their health status as unsatisfactory (OR, 95%CI 0.59, 0.42–0.82 comparing nonusers with those using the Internet daily). Finally, the use of the Internet was consistently associated with a lower prevalence of chronic conditions and disability, as well as less frequent visits to health care facilities.

**Conclusions:**

In conclusion, in contrast to earlier findings, Internet use is not associated with favourable patterns of lifestyle or higher self-rated health in older Polish adults. However, persons with chronic conditions or disabilities less frequently declare the use of the Internet. It was also revealed that older adults and the elderly who make use of the Internet are less likely to utilise health services.

## Introduction

The diffusion of information and communication technologies (ICT) is perceived as a determinant of human progress, which is understood to be the ability of every member of a society to live in an environment offering high economic, political, and civil liberties [1]. According to the International Development Union, ICT can help accelerate progress towards each of the 17 Sustainable Development Goals determined by the United Nations [2, 3]. However, the expectations that the Internet should be treated as an enabler of socioeconomic growth had been articulated much earlier [4, 5].

The growing role of the Internet in providing access to health-related information and health services during the COVID-19 pandemic has prompted some authors to recognise it as being as one of the key determinants of health [6]. Benda and Ancker believe that the COVID-19 pandemic has demonstrated that the lack of broadband access to the Internet has an influence on each of the social determinants of health identified by the American Medical Association [7]. According to these authors, it has a significant effect on the provision of health care services, economic stability, education, the distribution of food, the maintenance of social interactions and social contacts despite the demands for social distancing.

There are many reports indicating that the older members of society suffer from a significant digital divide [8–12]. According to the broad review prepared by Fang et al., the access and use of ICT, including the Internet, by older adults and the elderly is mediated by several factors, such as education, income, gender, disability status, place of residence and the person’s relationship status [13]. A study in the early 2000s revealed that greater age was not only associated with less frequent access to the Internet but also with a specific pattern of usage, including a more narrow range of personal goals pursued online and accessing a smaller number of sites [14].

However, there are also many reports suggesting that elderly Internet users demonstrate improved indicators of wellbeing, mental and physical health status and health behaviours when compared to nonusers. In addition, the use of the Internet was shown to be significantly associated with better self-rated health [15–17], higher life satisfaction [18–20], better quality of life [21], improved general or psychological wellbeing [18, 20, 22], decreased perceived loneliness [18], lower depression and/or anxiety [17, 19, 20, 23–27] and more favourable health behaviours [28, 29] as well as greater participation in preventative programmes [30, 31]. There are also reports suggesting that the use of ICT may be associated with a lower risk of dementia or at least with a slower rate of cognitive decline [26, 30, 32–34]. Some authors suggested a significant relationship between Internet use and social exclusion [35] or social capital [23].

It is not clear if using the Internet results in a more active approach to a person’s health management and more beneficial health behaviours, or if there is another factor, or factors, which lead to greater Internet use and more positive health-related outcomes. It seems that socioeconomic status and health literacy (HL) could be the common determinants. According to Yoon et al., the digital divide experienced by many older adults depended on variables related to socioeconomic status and ethnicity [36]. The study performed by Levy et al. suggested that elderly individuals with low HL used the Internet to obtain health information less frequently than those with adequate HL [37].

According to the projections presented by Eurostat, Poland will continue to experience an ageing society in the incoming decades [38]. This trend, as in many other European countries, is related to increasing life expectancy and low fertility rates [38]. In July 2020, the population 65 years and older comprised 17.7% of the Polish population [39]. It should also be noted that although in the last decade the number of Polish Internet users has been growing steadily, the percentage of older Internet users is still relatively low. In 2019, 69% of adults in Poland accessed the Internet at least once a week, but for the youngest adults (those below 35 years old), the percentage was nearly 100%. Of people aged 50–64 years old, 56% access the Internet, but only 26% of those 65 years and over [40]. The COVID-19 pandemic has greatly increased the interest in telemedicine and e-health by citizens, patients and health professionals, but telephone-based consultations still remain the most common type of telemedicine contact, especially for older people [41]. Earlier studies consistently showed that using the Internet for accessing health-related information in Poland correlated with younger age and that people aged 50 years or more relatively rarely used the Internet as a source of health-related information [42–44]. These findings are in line with the studies performed in other countries. The review carried out by Reiners et al. showed that older age, apart from lower education, living in rural areas or living alone, was associated with significantly lower use of e-health technologies [45].

If it were not for the digital divide, older people could gain considerable benefit by accessing health-related information and services through the Internet. It is obvious that the prevalence of chronic diseases increases with age and inevitably, multimorbidity becomes increasingly common in the elderly population. For those suffering from chronic diseases and disabilities, the personal difficulties could be alleviated by the knowledge and information about available services obtained from the Internet and related technologies. Some earlier studies have reported that people suffering from chronic conditions were likely to be more active Internet users. Bansil et al. reported that respondents suffering from a chronic disease were 1.3 times more likely to use the Internet to access health-related information than health people [46]. Using multiple regression analysis of the 2007 U.S. data on Internet use, Ayers & Kronenfeld reported that not merely the presence of a particular chronic illness, but rather the total number of chronic conditions determined the degree of Internet use [47]. Choi & DiNitto reported that people having more chronic conditions made 1.15 times greater use of the Internet for health-related tasks [48].

As it appears that the older segment of the Polish population does not make full use of the growing availability of the Internet, it was considered important to find if those browsing the internet are able to attain health-related benefits. Therefore, the main aim of this study was to make an assessment of the association between Internet use and health-related outcomes in a representative sample of the Polish population of adults 50 years and older. The effects of Internet use were analysed with multivariate models after adjusting for sociodemographic and economic factors. Key health-related outcomes addressed in the analysis included the self-assessment of health status, the prevalence of chronic conditions and disability, the utilisation of health services, health behaviours and HL.

## Methods

### Survey

The study is based on the data derived from a survey carried out in December 2017 using the computer-assisted telephone interviewing technique (CATI). The study sample (N = 1000) was representative of the Polish population aged 50 years and older. The survey was performed by the Biostat Sp. z o.o. (Rybnik, Poland), a company experienced in carrying out polls which was selected as a result of the obligatory procurement procedure in the University [49]. The respondents were recruited by the stratified proportional sampling of a database of mobile and stationary phone numbers developed by the Biostat Company. The structure of the sample corresponded to the Polish population for age, education, place of residence and NUTS1 region. The strata were based on data given in the 2016 Local Data Bank of Statistics Poland [50]. The size of the sample ensured that the degree of sampling error was no greater than 3.0%, assuming the population proportion 0.5 and a confidence level of 0.95.

The questionnaire used in the survey consisted of 58 items and included the Polish version of the tool for the assessment of HL (HLS-EU-Q16) [51]; the Polish version of the eHealth Literacy Scale (Pl-eHEALS) [52], and a set of items asking about the use of the Internet, the individual’s health behaviours, the self-assessment of health status, the prevalence of chronic diseases, any disability status, and the utilisation of the health care services. In addition, questions asking about sociodemographic characteristics were included. HLS-EU-Q16 is a short, 16-item version of the questionnaire developed by the European Health Literacy Survey Project team [51]. Pl-eHealth consists of 8-item requiring self-assessment of skills needed to understand and use health-related information obtainable online [52]. The use of the Internet, self-assessment of health status, the prevalence of chronic diseases, disability status and utilisation of the health care services were assessed by a single response. Additionally, specific health behaviours were assessed by single items to which a response could be given according to a relevant frequently scale.,

This study was approved by the Bioethical Committee of Jagiellonian University (No. 122.6120.313.2016 issued 24 November 2016). The respondents were informed about the aims of the study, and only those giving their consent participated in the survey.

### Statistical analysis

IBM SPSS v.25 software (IBM Corp. Armonk, NY, USA) was applied for the statistical analysis of data. Descriptive statistics were computed for categorical and continuous variables. The level of statistical significance was established at the level < 0.05.

The assessment of the association between Internet use and dependent variables was carried out with uni- and multivariate logistic regression models after adjusting for the sociodemographic variables. The dependent variables were derived, after any necessary dichotomisation from the responses to items asking about smoking, consumption of alcoholic beverages, undertaking physical exercise, the consumption of fruit and vegetables, the self-assessment of health status, the prevalence of chronic diseases and disabilities, visits to health care facilities and hospitalisations in preceding 12 months and HL. The body mass index (BMI) based on the height and weight reported by the respondents was also calculated and categorised for comparisons between those having normal weight or being underweight (< 25 kg/m2) and those being overweight or obese (25 kg/m2 and above).

The HL score was calculated on the basis of the responses given to the 16 items of the HLS-EU-Q16, which ranged from very difficult to very easy with an option “difficult to say/not applicable” [51]. The responses very difficult and difficult were scored 0, very easy and easy 1. The response “difficult to say/not applicable” was treated as a missing value [51]. The HL score was calculated as the sum of individual scores only if the number of missing values was less than 3. A score below 9 was assessed as having “inadequate HL”, 9–12 as possessing “problematic HL” and over 12 points as “sufficient HL”.

In the multivariate logistic regression model, the effect of Internet use on dependent variables was calculated after adjusting for socioeconomic variables: age, gender, place of residence (3 categories), level of education (3 categories), marital status (2 categories), vocational status (3 categories) and net income per person in the household (4 categories, including a separate category for those who refused to reveal their income). The variable reflecting Internet use were given four values coded as 0 – “no use”, 1 – “a few times a month or less”, 2 – “a few times a week”, and 3 – “every day”. The category “no use” was a referential category applied in regression modelling.

Before the multivariate logistic regression models were developed, the multicollinearity was assessed. The variance inflation factor and the tolerance were in the expected ranges for all models. For each multivariate regression model, the Hosmer and Lemeshow chi^2^ test and the Nagelkerke R^2^ were calculated. For the independent variables included in the logistic regression models, the p value, the odds ratio (OR) and 95% confidence interval (95%CI) are reported.

## Results

### Characteristics of the study group

The age structure of the study group corresponded to the age distribution of the population of people 50 years or older in Poland in 2016. The most numerous strata consisted of respondents aged 55–59 (21.1%), those 60–64 years (19.2%) and 50–54 years old (18.2%). The proportion of females was 55.8%. In the study sample, 26.3% of the respondents had attained a University, and 27.8% had a lower than secondary education. Residing in a rural area was indicated by 27.2% of the participants, and 22.5% lived in an urban area with a population of at least 200,000. Those retired, on a disability pension or vocationally inactive persons made up 71.7% of the group. The percentage of married persons was 71.9%. The proportion of respondents who did not use the Internet was 51.1%, but a significant proportion of the group, 26.2%, accessed the Internet every day. Of those for whom the HL score could be calculated, a sufficient level of HL was found in 55.0% of respondents. However, as many as 36.9% of the respondents were unable to provide a number of valid responses to the HLS-EU-Q16 questionnaire required for determining the HL score. Among other important findings, 73.3% of respondents were overweight or obese, 72.6% confirmed that they suffer from at least one chronic condition, and 19.7% indicated that they have a disability. Detailed characteristics of the study sample are shown in Table 1.

**Table 1:** Characteristics of the study group

Variable	Categories of variable	%	N
Age	50–54	18.2	182
	55–59	21.1	211
	60–64	19.2	192
	65–69	9.3	93
	70–74	13	130
	> 75	16.7	167
Gender	Female	55.8	558
	Male	44.2	442
Education level	Lower than secondary	27.8	278
	Secondary or post-secondary non-tertiary	45.9	459
	University	26.3	263
Place of residence	Rural	27.2	272
	Urban < 20,000	18.1	181
	Urban 20,000 to < 100,000	16.8	168
	Urban 100,000 to < 200,000	15.4	154
	Urban 200,000 to 500,000	11.0	110
	Urban > 500,000	11.5	115
Vocational status	Employee of public or private sector	16.7	167
	Entrepreneur or farmer	19.7	197
	Retired, on disability pension or vocationally inactive	71.7	717
Net income per household member	< 1500	31.3	313
	1500–3500	29.4	294
	> 3500	17.3	173
	Refused to reveal	22.0	220
Marital status	Married	71.9	719
	Single, widowed, divorced or separated	28.1	281
Internet use	No use	51.1	511
	A few times a month or less	7.9	79
	A few times a week	14.8	148
	Every day	26.2	262
Smoking	No	80.7	805
	Yes	19.2	192
Alcohol consumption	No	66.0	589
	Yes	34.0	304
	Refused to reveal	10.7	107
Physical activity	Less often than several times a week	30.6	259
	At least several times a week	69.4	587
The consumption of fruit and vegetables	Less often than once a day	13.1	128
	At least once a day	86.9	848
Self-assessment of health status	Unsatisfactory	32.1	321
	At least satisfactory	67.9	679
Health literacy	Inadequate or problematic	45.0	347
	Sufficient	55.0	284
Chronic condition	No	27.4	265
	At least one	72.6	702
Disability	No	80.3	793
	Yes	19.7	194
Weight	Normal or underweight	26.7	267
	Overweight or obese	73.3	733
Visit to health care facility in a preceding year	No or less than 3 times	23.1	237
	At least 3 times	76.1	753
Hospital admission in a preceding year	No	78.8	788
	Yes	21.2	212

### Internet use as a predictor of health-related outcomes

The results of uni- and multivariate logistic regression modelling are shown in Table 2. Univariate logistic regression modelling revealed that Internet users more frequently consume alcoholic beverages (OR, 95%CI 1.68, 1.12—2.51 for comparison between nonusers vs those who use the Internet a few times a week) and less often undertake physical activity (OR, 95%CI 0.52, 0.30—0.90 for nonusers vs respondents using the Internet several times a week). Those using the Internet a few time a week, or every day, were about 40% more likely to rate their health as unsatisfactory than nonusers (OR, 95%CI 0.59, 0.40—0.89, and 0.57, 0.41—0.79, respectively). Persons suffering from chronic disease or with disabilities were also less likely to use the Internet. Internet users were 70–80% less likely to confirm a chronic condition than nonusers. The odds that a daily Internet user is a person with a disability was about 60% lower than that of a nonuser (OR, 95%CI 0,37, 0.24 – 0.57). The users of the Internet less frequently visited health care facilities or had been admitted to hospital in the preceding year. Interestingly, those using the Internet only rarely were more likely to have inadequate or problematic HL than the nonusers (OR, 95%CI 0.73, 0.54 – 0.99 for comparison of nonusers those vs the respondents using the Internet not more often than a few times a week). However, there was no difference in the prevalence of sufficient HL between nonusers and the more frequent users of the Internet.

**Table 2:** Uni- and multivariate logistic regression models for health-related outcomes in relation to Internet use

Dependent variable	Internet* use	OR	95%CI	p^&^	aOR	95%CI	p^#^
Smoking	1	0.99	0.67–1.44	0.94	0.69	0.35–1.36	0.284
	2	0.69	0.34–1.39	0.30	1.35	0.86–2.12	0.19
	3	1.33	0.81–2.16	0.26	1.03	0.7–1.52	0.883
Alcohol consumption	1	1.64	0.99–2.72	0.055	1.67	0.99–2.79	0.05
	2	1.68	1.12–2.51	0.012	1.65	1.09–2.50	0.019
	3	1.44	1.03–2.01	0.033	1.41	1.00–1.98	0.05
Physical activity	1	1.01	0.71–1.44	0.96	0.48	0.28–0.83	0.008
	2	0.52	0.30–0.90	0.020	0.99	0.64–1.57	0.992
	3	1.07	0.67–1.72	0.77	0.94	0.65–1.35	0.725
Consumption of fruit and vegetables	1	1.48	0.96–2.27	0.076	0.87	0.42–1.8	0.702
	2	1.34	0.64–2.82	0.44	0.85	0.47–1.51	0.568
	3	1.33	0.73–2.41	0.35	0.64	0.42–0.99	0.049
Self-assessment of health status	1	0.68	0.41–1.15	0.15	0.7	0.41–1.19	0.19
	2	0.59	0.40–0.89	0.012	0.58	0.38–0.88	0.010
	3	0.57	0.41–0.79	0.001	0.59	0.42–0.82	0.002
Health literacy	1	0.73	0.54–0.99	0.047	0.71	0.52–0.97	0.034
	2	0.59	0.34–1.02	0.057	0.62	0.35–1.07	0.087
	3	0.90	0.59–1.36	0.61	0.86	0.56–1.31	0.48
BMI	1	1.18	0.84–1.65	0.34	0.89	0.51–1.56	0.69
	2	1.07	0.61–1.88	0.82	0.69	0.45–1.05	0.079
	3	0.83	0.54–1.29	0.41	0.91	0.64–1.29	0.58
Chronic condition	1	0.30	0.16–0.48	< 0.001	0.27	0.16–0.47	< 0.001
	2	0.22	0.16–0.33	< 0.001	0.21	0.13–0.32	< 0.001
	3	0.21	0.15–0.30	< 0.001	0.2	0.14–0.29	< 0.001
Disability^%^	1	0.74	0.50–1.09	0.13	0.73	0.49–1.09	0.12
	2	0.37	0.24–0.57	< 0.001	0.35	0.22–0.54	< 0.001
Hospital admissions in preceding year	1	0.76	0.53–1.07	0.12	1.04	0.58–1.88	0.888
	2	0.78	0.43–1.43	0.43	0.6	0.35–1.02	0.057
	3	0.45	0.26–0.77	0.004	1.32	0.92–1.89	0.13
Visits to health care facility in preceding year	1	1.59	1.13–2.25	0.008	0.62	0.36–1.07	0.088
	2	0.92	0.53–1.60	0.78	0.76	0.48–1.19	0.22
	3	1.22	0.77–1.94	0.40	0.65	0.45–0.93	0.017

*Categories of Internet use: referential category of Internet use used in logistic regression modelling—“no use”, 1— “a few times a month or less often”, 2— “a few times a week”, 3— “everyday”; ^%^–due to low frequencies of selected categories of Internet use, the categories were collapsed: 1—not more than a few times a week, 2—every day, referential category— “no use”, ^&^p value for univariate logistic regression model; ^#^p value for univariate logistic regression model; OR—odds ration; 95%CI—95% confidence interval; aOR—odds ration after adjusting for sociodemographic variables (age—as a continuous variable, gender, education level, place of residence, marital status, vocational status, net income per household member), BMI—body mass index

After adjusting for sociodemographic variables, the association between the use of the Internet and alcohol consumption was maintained (OR, 95%CI 1.65, 1.09 – 2.5). The association between more frequent Internet use and lower physical activity was maintained, but it was significant only when comparing Internet nonusers and those who used it a few times a month or less frequently (OR, 95%CI 0.48, 0.28–0.83). There was no significant difference between nonusers and those using the Internet a few time a week or every day. The adjusted model revealed that respondents who used the Internet most frequently consumed fruit and vegetables less often than nonusers (OR, 95%CI 0.64, 0.42–0.999).

The multivariate model showed that persons who use the Internet not more than a few times monthly are more likely to have inadequate or problematic HL than nonuser (OR, 95%CI 0.71, 052–0.97). Nevertheless, the prevalence of insufficient HL did not differ between nonusers and those using the Internet daily or a few times a week.

Consistently, the use of the Internet, when compared with non-usage, was significantly associated with a lower prevalence of chronic diseases. Internet users were about 70%-80% less likely to report chronic disease than nonusers. It could be also seen that for those who used the Internet every day, the percentage reporting disabilities was about 65% lower than the nonusers (OR, 95%CI 0.34, 0.22–0.54).

As for the utilisation of health care resources, after adjusting for socioeconomic variables, a significant association was found only for visits to health care facilities but not for hospitalisations in a preceding year. Daily Internet users had less frequently visited health care facilities in the previous 12 months than nonusers (OR, 95%CI 0.65, 0.45–0.93).

## Discussion

### Main findings

In the study group, only about 49% were users of the Internet. This is significantly lower than in the general population [53]. It appears that after adjusting for socioeconomic variables, the use of the Internet was significantly associated with unfavourable health behaviours. However, this relationship was observed only for particular categories of the frequency of Internet use. The respondents who accessed the Internet a few times weekly had an increased likelihood of consuming alcoholic beverages more frequently. The respondents who used the Internet not more frequently than a few time a month less frequently undertook physical activity. Finally, frequent users of the Internet consumed less fruit and vegetables than nonusers.

### Heath behaviours

A negative association between Internet use and the health behaviours reported here is not supported by other studies. Xavier et al. analysed the data derived from 5,900 respondents aged 50 years or older from Waves 1 to 5 of the English Longitudinal Study of Aging (ELSA) [30]. They found that Internet use was consistently associated with a weekly period of moderate to vigorous physical activity, more frequent daily consumption of at least five portions of fruit and vegetables and less frequent smoking. The degree of participation in preventative programmes was not assessed in the Polish sample. Xavier et al. also reported that the use of the Internet was positively associated with taking part in the colorectal cancer screening programme.

Contrary findings to those obtained from the Polish sample of older adults and elderly were also reported for a sample of adults from Taiwan by Peng & Chan [29]. They found that persons 40 years and older recruited to the 2011 wave of the Taiwan Social Change Survey who used the Internet regularly were more likely to follow an overall healthy lifestyle (OR, 95%CI for the combined lifestyle indicator: 2.20, 1.28–3.78). The regular Internet users, after adjusting for sociodemographic variables, more frequently reported that they engaged in physical exercises and the regular consumption of fruit and vegetables, and less often smoked tobacco or chewed betel nuts. However, for those reporting heavy drinking of alcohol, there was no significant association with their use of the Internet.

Interesting results on the relationship between Internet use and the involvement of couples in preventative activities were described by Nam et al. [31]. They analysed the data of the survey undertaken on a sample of 5,143 pairs of coupled individuals from the 2010 and 2012 waves of the Health and Retirement Study in the USA. They found that Internet use was related to a higher likelihood of husbands undergoing prostate examinations and cholesterol tests. For wives, the use of the Internet was associated with an increased likelihood of accepting flu shots and their husbands’ receiving prostate examinations. Interestingly, husbands’ Internet use was not related to wives’ preventative health behaviours.

### Self-assessment of health status

The older Polish adults who used the Internet were also more critical when assessing their health status. Those who used the Internet at least a few times a week assessed their health as being significantly worse than nonusers. Earlier studies presented contrary results. The univariate analysis performed by Gracia & Herrero on the data from a survey on the Spanish adult population, 55–74 years old, revealed that Internet use was associated with better self-rated health [15]. However, the Internet effect disappeared when social class was introduced to the model. According to Lee et al., greater use of communication technologies, including e-mailing and texting by participants in the 2011 wave of the USNHIS, was significantly associated with a lower risk of severe depressive symptoms and a higher level of self-rated health [17]. Falk-Erhag et al. observed a significant association between more frequent Internet use and a good self-rating of health in a population-based sample of 70-year-olds participating in the Gothenburg H70 Birth Cohort Study (n = 1136). However, the authors remarked that Internet use was of minor importance to the self-rated health of older adults and the inclusion of health-related variables to the model considerably increased its explanatory power [54].

### Health literacy

The Polish study showed that rare Internet use was associated with the prevalence of inadequate or problematic HL. The difference in HL was observed only between nonusers and those who used the Internet not more frequently than a few times a month. They were 30% more likely to have problematic or inadequate HL. There were no differences in the level of HL between the nonusers and those using the Internet more frequently than a few times a month. This finding is in agreement with the study on the general Polish population, which has not shown a significant association between Internet use and the prevalence of limited HL [55]. The results of studies undertaken in other countries are not unequivocal; however, most studies tend to suggest that Internet use is associated with higher HL. Wister et al. observed that computer and Internet use, apart from other learning enabling factors, were associated with better HL among older adults in Canada [56]. Using the Internet was rarely reported by older adults possessing lower levels of HL in the study carried out by Echt & Burridge [57]. The study of Levy et al. [37] showed that elderly people with low HL were less likely to use the Internet, and those who did use the Internet, were less likely to use it to get health or medical information. The analysis performed by Jiang & Beaudoin on data originating from the 2013 edition of Health Information National Trends Survey (HINTS) in the USA showed that in the sample of respondents with a mean age of 51.2 years, greater health-related use of the Internet was associated with higher HL [58]. Unfortunately, in this study, the HL of Internet users and nonusers was not compared. Kobayashi et al. analysed data from the English Longitudinal Study of Ageing from years 2004–2011 [59]. They found that consistent Internet use had a protective effect against a decline in HL with ageing. A strong association between HL and Internet access and use was found by Estacio et al., who performed a city-wide HL survey in England [60]. Interestingly, the mean HL score was higher both for older adults receiving more health-related information from the Internet and health providers than for those who less often used these sources [61]. There are also studies that did not confirm a significant association between Internet use and the level of HL among older adults and the elderly. Gutierrez et al. did not show a significant relationship between Internet use and health literacy in patients attending two clinics in Dallas, TX [62]. Another study, on a group of patients with heart failure, revealed that higher HL was associated with the use of e-mail but not with Internet use [63]. Some authors suggested that even if the Internet became an important source of information for patients, it is poorly adapted to serve as a tool for health-related decision making. In addition, the Internet can actually be a source of problems for one’s HL when inaccurate online information is accessed by patients [64].

### Chronic conditions

The Polish study revealed that persons with at least one chronic condition were more frequently nonusers of the Internet, whereas those who used the Internet were about 70–80% less likely to suffer from chronic disease. It is not fully clear if the use of the Internet may have a protective effect against the prevalence of chronic diseases or whether persons who do not suffer from chronic conditions are more eager to use the Internet. The results reported are contrary to the observations reported by other researchers. According to Bansil et al., people with chronic disease were 1.3 times more likely to use the Internet to access health-related information [46]. Ayers & Kronenfeld suggested that the total number of chronic conditions determines the degree of Internet usage [47]. In their opinion, more frequent accessing online health information is associated with the likelihood of improving one’s health behaviours. A 15% increase of health-related Internet use by persons with chronic diseases was also reported by Choi & DiNitto [48], and similar results were reported for a sample of adults 40 years and older from Taiwan [29]. The assumption that having a diagnosed chronic disease should be a prompt for searching for health-related information and the motivation to use the Internet for this purpose is convincing. Why such a relationship is not found in Polish the older adult and elderly Polish population, is not clear. However, earlier studies assessing the determinants of the use of health information available online consistently showed that age played a key role and that older adults much less frequently used the Internet for this purpose [42–44]. Furthermore, the 2015 study showed that older people suffering from chronic conditions in Poland were less likely to accept ehealth solutions than younger patients [65]. The fact that older patients with chronic diseases in Poland rarely search for health information on the Internet may suggest that physicians are still the main source of such information for these people. In effect, there is no increase in Internet use by older groups in relation to their chronic conditions. One could also consider the hypothesis that Internet use may have some protective effect against the prevalence of chronic diseases, but the association found between Internet use and unfavourable health behaviours seems to contradict it.

### Disability

It is obvious that persons with disabilities are prone to a significant digital divide. Many studies have evidenced that persons with disabilities experience a meaningful digital divide [66–69]. It may be that the digital divide is the consequence of the less favourable socioeconomic situation resulting from the disability rather than the disability itself [70]. Therefore, it is not surprising that in the reported study, older adults and elderly persons with disabilities used the Internet less frequently than those without disabilities. However, for persons with disabilities, the use of the Internet may be associated with improved wellbeing and health-related measures, as has been reported earlier [71].

### The utilisation of health care resources

The Polish study revealed that respondents accessing the Internet every day, less frequently visited health care facilities. After adjusting for sociodemographic variables in the study group, there was no significant association between Internet use and hospital admissions. Earlier studies have not provided consistent results on the association between Internet use and utilisation of health services. Deetjen & Powell found that Internet use by the general population was associated with lower use of health services [72]. However, the analysis of data obtained from participants in the Health and Retirement Study conducted in the USA revealed that the use of online health information was positively associated with doctor’s visits at the time of the survey but not two years previously [73]. Furthermore, patients with diabetes using such information had significantly fewer doctor visits than nonusers at both time points. Contrary results were reported by Narcisse et al. [74]. Their survey carried out on Native Hawaiians and Pacific Islanders showed that the use of health-related online information increased the odds of a person visiting emergency departments or using outpatient services. Finally, Hansen et al. reported that greater age but not the use of health-related online resources was significantly associated with visits to a general practitioner’s surgery [75].

### Variables not addressed in the current study

There are many other outcomes, such as mental health, cognitive status, life satisfaction, quality of life, social inclusion, loneliness, and social or psychological capital, that were not analysed in this study. Several authors consistently described the relationship between Internet use and the lower prevalence of depression in older populations. Choi & DiNitto analysed the data from the National Health and Aging Trends Study based on a nationally representative sample of the beneficiaries of U.S. Medicare aged 65 years or above [48]. They found that Internet use was negatively associated with the symptoms of depression and anxiety and measures of psychological capital but positively with most measures of social capital. The analysis of data obtained by the China Family Panel Study in 2016, after adjustment for sociodemographic variables, physical health, life satisfaction and intelligence level, showed that older adults who use the Internet demonstrate lower levels of depression than those who did not use the Internet [24]. Liao et al. analysed data provided by 18,492 respondents aged 45 years and older participating in China Health and Retirement Longitudinal Study (CHARLS) 2015 living in urban and or rural areas in China. They found that occasional use of the Internet or one of the types of digital device was associated with the respondents living in rural areas having a lower risk of experiencing depression than Internet users living in urban areas [25]. According to the study of Heo et al. [18] carried out on data from the 2008 edition of the U.S. Health and Retirement Study, greater use of the Internet was a significant predictor of higher social support, decreased loneliness, better life satisfaction and psychological wellbeing for people aged 65 years and above. Having analysed the data from 4 waves of the Understanding Society, the U.K Household Longitudinal Study 2009–2013, Sacker et al. reported in 2017 that the use of the Internet was one of the moderators of the association between poor health and social exclusion in those aged 65 or older [35]. According to Lifshitz et al., four functions of Internet use: interpersonal communication, information seeking, task performance, leisure and recreation, were correlated with life satisfaction in people 50 years and older [20]. Furthermore, task performance and leisure activities online were negatively correlated with depression. However, after controlling for sociodemographic variables, only leisure activities on the Internet were still significantly associated with measures of wellbeing.

Lam et al. reported that higher life satisfaction, apart from a lower depression, was found in Internet users who were participants of the Waves 6 to 8 of the English Longitudinal Study of Ageing [19]. Boz et Karatas undertook a review of the studies on the relationship between the use of the Internet and the quality of life of elderly people published after 1990. They concluded that functional use of a computer and the Internet is associated with improved QoL for people in this group [21].

### Limitations

The first limitation of the reported study is related to its design. Unfortunately, a cross-sectional study does not allow the cause-effect dependencies to be traced. Therefore, the results presented here do not respond to the key question of whether the Internet is a real facilitator of beneficial health outcomes of older adults. Additionally, a wide range of health-related outcomes were assessed as dependent variables as they were possibly associated with Internet use. However, the nature of their relationships with using the Internet could be more complex. The regression models developed in the analysis were adjusted for key sociodemographic and economic factors, but there is a substantial difference in the use of the Internet between the older adults 50–65  years old and those aged more than 65. According to the 2019 survey, 56% of the 50–65 years old make use of the Internet, but for the over 65s, there are only 26% of users [40].

## Conclusions

Contrary to the expectations, the use of the Internet was not positively associated with favourable health behaviours by those aged 50 years and older. Internet users were likely to drink alcoholic beverages more frequently. They were also less likely to participate in physical activities or to consume fruit and vegetables. No statistically significant association was observed between Internet use and smoking. Interestingly, Internet users were more inclined to assess their health status as unsatisfactory than nonusers. Furthermore, persons suffering from more chronic conditions were less likely to use the Internet. Contrary associations, both for health behaviours, self-rated health and the prevalence of chronic conditions, have been reported by other authors. However, users of the Internet less frequently utilised health care services. As for HL, those rarely using the Internet had a lower level of HL than nonusers, but there was no difference in the HL score between the more frequent Internet users and the nonusers.

A more detailed study of the results indicates that the differences between the users and nonusers of the Internet were not consistent for all Internet users. Frequently, differences were observed between nonusers and only one of the frequency categories of Internet use. For example, lower HL and less often undertaking physical activity in comparison to nonusers was observed only for those respondents who used the Internet most rarely. In turn, a difference in the consumption of fruit and vegetables was seen only between those who used the Internet daily and nonusers. It is evident that older users of the Internet are not a homogenous group.

The results of this study encourage further research on the association between Internet use and health-related outcome in the older strata of Polish society. It would be interesting to investigate the reasons why less favourable health behaviours appear to be related to the use of the Internet because Internet users have greater access to up-to-date guidelines to ensure a healthy lifestyle.

It is obvious that relationships between Internet use and outcomes related to health are the result of complex interactions. Consequently, a cross-sectional study does not allow for any reasoning or determination of the causative pathways. It also seems that the use of the Internet may also be an antecedent or a consequence of different types of such variables. The causative reasons for the differences in the observed relationships between the Polish and other populations would also need a more detailed study.

## Data Availability

The data set used for the analysis in the study is available from the corresponding Author on reasonable request.

## Abbreviations

95%CI: 95% Confidence interval
aOR: Adjusted odds ratio
BMI: Body mass index
CATI: Computer-assisted telephone interviewing technique
HL: Health literacy
HLS-EU: European Health Literacy Survey
ICT: Information and communication technologies
OR: Odds ratio
Pl-eHEALS: Polish version of the eHealth Literacy Scale
